# Supplementary material for: TIM4 expression by dendritic cells mediates uptake of tumor-associated antigens and anti-tumor responses
Source: Nat Commun. 2021 Apr 14;12:2237. doi: 10.1038/s41467-021-22535-z (PMC8046802; doi:10.1038/s41467-021-22535-z)
Supplement: Supplementary file 1 — Supplementary Information [file 41467_2021_22535_MOESM1_ESM.pdf]

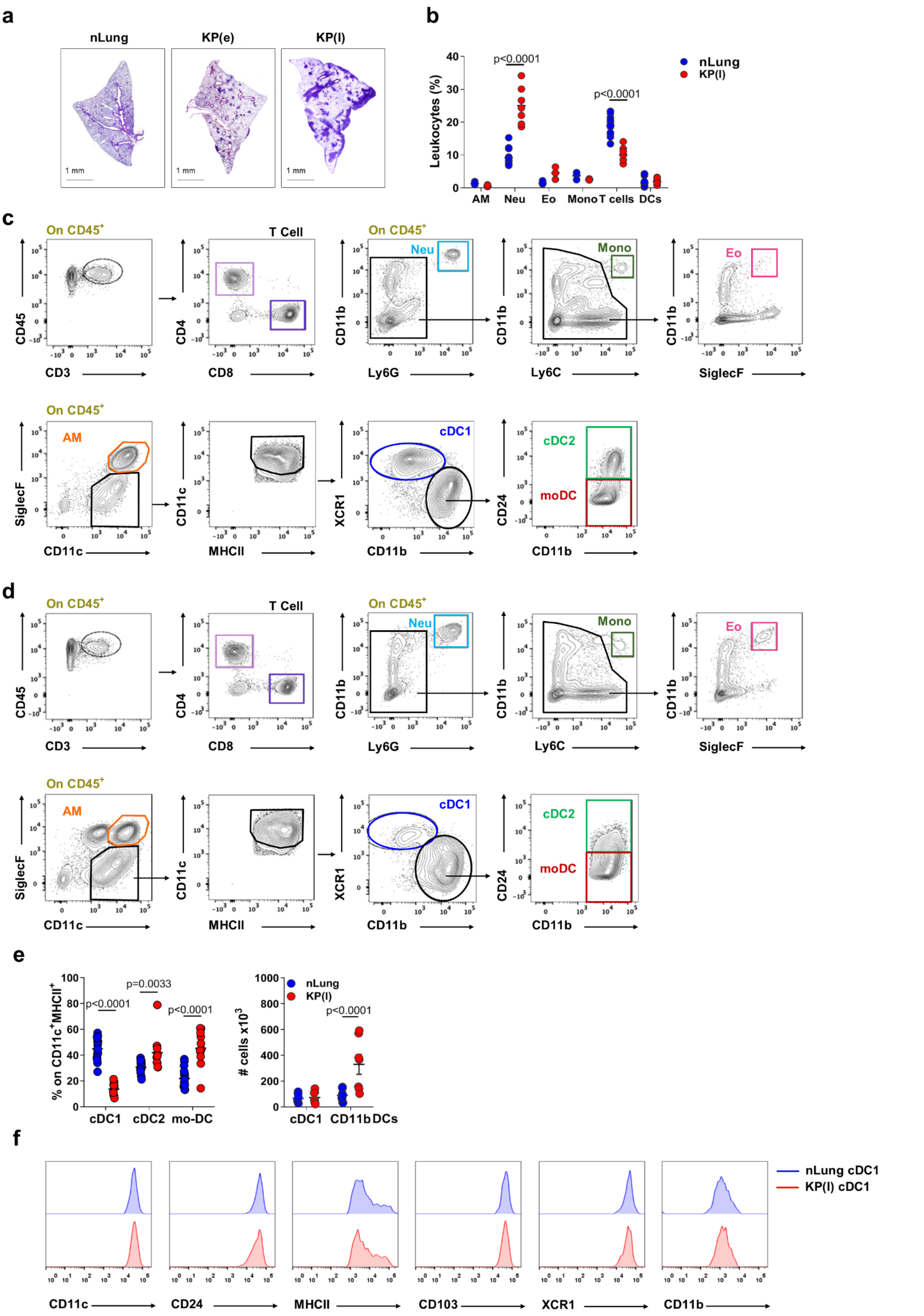

a
nLung
KP(e)
KP(l)
1 mm
b
p<0.0001
p<0.0001
Leukocytes (%)
AM
Neu
Eo
Mono
T cells
DCs
nLung
KP(l)
c
On CD45+
CD45
CD3
T Cell
CD4
CD8
Neu
CD11b
Ly6G
Mono
Ly6C
Eo
SiglecF
AM
CD11c
MHCII
cDC1
XCR1
cDC2
moDC
CD24
d
e
% on CD11c+MHCII+
p<0.0001
p=0.0033
cDC1
cDC2
mo-DC
cells x10³
cDC1
CD11b DCs
f
nLung cDC1
KP(l) cDC1
CD103

Supplementary Figure 1. Tumor-infiltrating leukocytes and cDC1 profiling in KP bearing lungs.

a) Representative hematoxylin/eosin sections illustrating nodules in PBS injected mice (nLung), early and late KP-bearing lungs. Scale bar 1mm. **b)** Relative abundance of immune subsets (AM, Alveolar macrophages; Neu, neutrophils; Eo, eosinophils; Mono, monocytes) in nLung or late KP-bearing lung tumors. Data represent 2 independent experiments. nLung: AM n=4, Neu=17, Eo n=3, Mono n=3, T cells n=16 and DCs n=17. KP(l): AM n=10, Neu=17, Eo n=3, Mono n=3, T cells n=16 and DCs n=17. **c-d)** Gating strategy used for the identification of leukocytes populations in nLung (**c**) and KP-bearing lungs (**d**). **e)** Frequencies of lung resident cDC1, cDC2 and monocyte-derived DCs (moDCs) in nLung and late KP tumors. Absolute numbers of cDC1 and $CD11b^+$ DCs in late KP tumors. Pooled data from 3 independent experiments. nLung n=20 or 11, KP(l) n=12 or 7. **f)** Representative histograms of lineage differentiation and maturation markers on cDC1 in nLung or late KP tumors. All data are expressed as mean ± SEM and significance was assessed by two-ways ANOVA followed by Sidak's post-test. Raw data and statistical analysis are available in source data file.

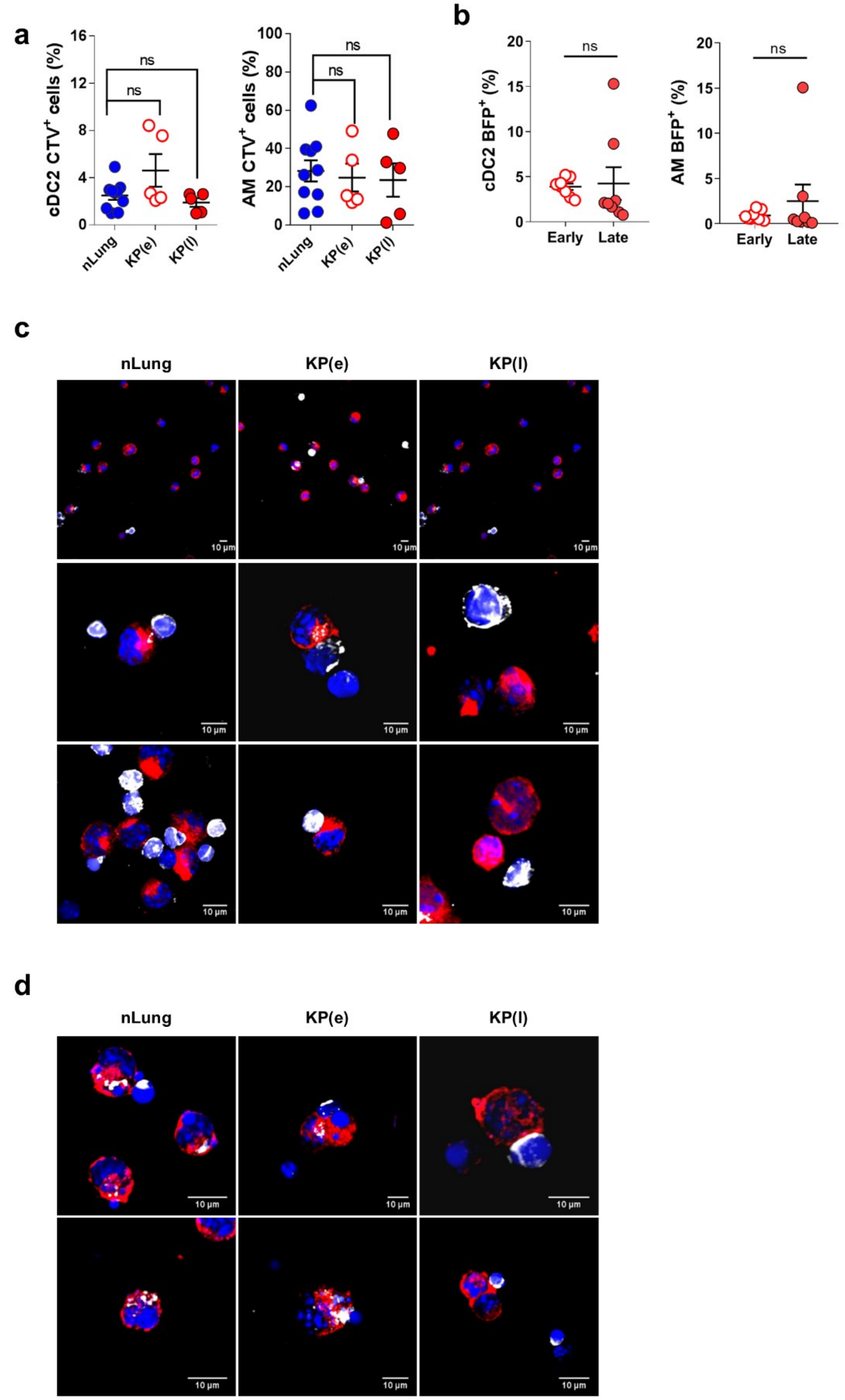

Supplementary Figure 2. Uptake of apoptotic thymocytes and tumor cells by lung phagocytes along tumor progression.

a) Relative to Fig 1a. nLung, early and late KP tumor-bearing mice were inoculated i.t. with CTV-labeled apoptotic thymocytes. The amount of fluorescence associated to cDC2 and alveolar macrophages (AM) was evaluated 2hrs post injection. nLung n=8, KP(e) n=5, KP(l) n=4. Significance was assessed by two-ways ANOVA followed by Sidak's post-test. **b)** Relative to Fig 1b. Quantification of BFP fluorescence associated to cDC2 and AM in lungs bearing early or late KP-BFP tumors. Data are pooled from two experiments with n=8 mice/group, two-tailed unpaired *t*-test. **c)** Relative to Fig 1c. Low and high magnification images as examples of binding of apoptotic thymocytes to cDC1 from control, early or late tumors. **d)** Relative to Fig 1d. Representative high magnification micrographs of uptake of apoptotic cells after 2hrs of incubation. All data are expressed as mean ± SEM. Raw data and statistical analysis are available in source data file.

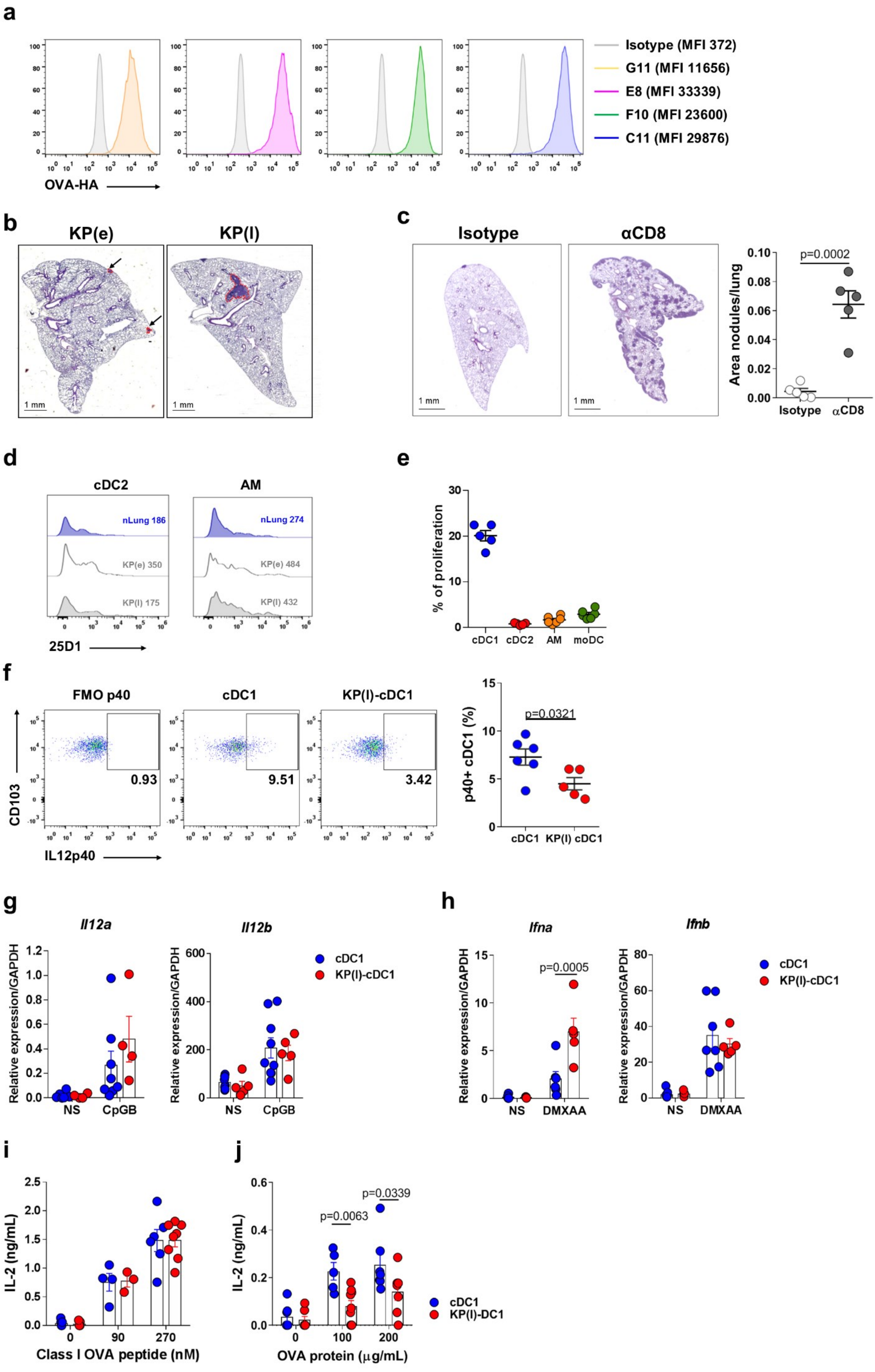
a
Isotype (MFI 372)
G11 (MFI 11656)
E8 (MFI 33339)
F10 (MFI 23600)
C11 (MFI 29876)
OVA-HA
b
KP(e)
KP(l)
1 mm
c
Isotype
αCD8
p=0.0002
Area nodules/lung
d
cDC2
AM
nLung 186
KP(e) 350
KP(l) 175
nLung 274
KP(e) 484
KP(l) 432
25D1
e
% of proliferation
cDC1
cDC2
AM
moDC
f
FMO p40
cDC1
KP(l)-cDC1
0.93
9.51
3.42
CD103
IL12p40
p40+ cDC1 (%)
p=0.0321
KP(l) cDC1
g
Il12a
Il12b
Relative expression/GAPDH
NS
CpGB
cDC1
KP(l)-cDC1
h
Ifna
Ifnb
p=0.0005
DMXAA
i
IL-2 (ng/mL)
Class I OVA peptide (nM)
j
p=0.0063
p=0.0339
OVA protein (μg/mL)
KP(l)-DC1

Supplementary Figure 3. Characterization of KP-OVA tumors, controls for cross-presentation of KP-OVA *in vivo*, cytokine production and cross-presentation of soluble antigens delivered *ex vivo*.

a) KP cells were transduced with a lentiviral vector expressing HA-OVA. The population was subcloned and HA-OVA expression in individual clones was evaluated by intracellular flow-cytometry. Clone G11 showing the lowest expression was selected for subsequent experiments. **b)** Hematoxylin/eosin staining of lung sections from KP-OVA early or late tumors. **c)** Mice were treated with anti CD8 depleting antibodies during challenge with KP-OVA tumors. Lungs were harvested two weeks after challenge for analysis of tumor burden. Representative hematoxylin/eosin staining shows nodules from isotype controls and CD8 depleted mice (5 mice/group). Quantification of nodules was calculated semi-automatically on 3 sections of the left lobe/animal. Significance was assessed by two-tailed unpaired *t*-test. **d)** Relative to Fig 1e. Controls of staining specificity by OVA-peptide:MHC-I antibody 25D1.16. Representative histograms show labeling in cDC2 and AM in nLung, early and late KP-OVA tumor bearing lungs. Peaks are overlaid with the corresponding isotype control. **e)** Cross-presentation by phagocytes in KP-OVA tumors. cDC1, cDC2, AM and moDCs were sorted from KP-OVA tumors and plated with CFSE-labeled OT-I cells *ex vivo*. The extent of proliferation induced by each subset is plotted as percentage of cells undergoing at least two proliferation cycles. One representative of three independent experiments is plotted, cDC1 n=5, cDC2 n=5, AM n=6, moDC n=6. **f)** *In vivo* production of IL-12p40 by nLung cDC1 and late KP tumor cDC1 was assessed by intracellular cytokine detection. Significance was assessed by two-tailed unpaired *t*-test of two independent experiments, cDC1 n=6, KP(l)-cDC1 n=5. **g, h)** cDC1 from nLung or late KP-bearing lungs were isolated by cell sorting and stimulated *ex vivo* for 3 hrs with TLR9 (CpGB) and STING agonists (DMXAA). Transcript expression of *Il12a*/*Il12b* (**g**) and *Ifna*/*Ifnb* (**h**) were evaluated by qPCR. Data in **g** cDC1 n=8 and KP(l)-cDC1 n=5, data in **h** cDC1 n=7 and KP(l)-cDC1 n=5, from 4 independent experiments each with pool of 3 mice/group. Significance was assessed by two-ways ANOVA followed by Sidak's post-test. **i, j)** cDC1 from nLung or KP late tumor bearing lungs were pulsed *ex vivo* with OVA class I peptide (**i**) or OVA protein (**j**) at different doses and co-incubated with OT-I. IL-2 was quantified by ELISA upon 16 hrs of co-culture. Each dot represents the pool of three mice from three independent experiments. Significance was assessed by two-ways ANOVA followed by Sidak's post-test. All data are expressed as mean ± SEM. Raw data and statistical analysis are available in source data file.

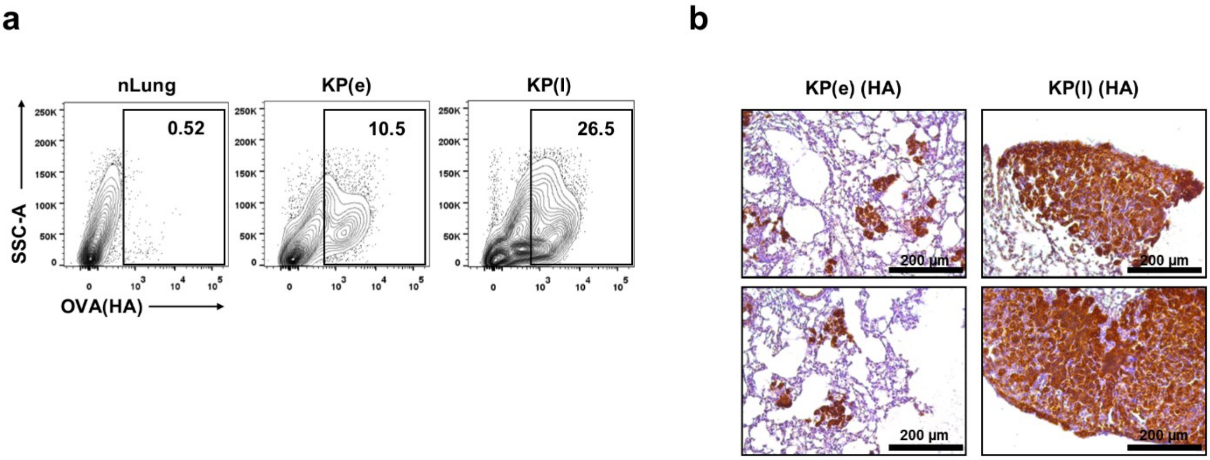

Supplementary Figure 4. Expression of OVA antigen is maintained in late KP-OVA tumors.
a, b) OVA expression in early and late KP-OVA tumors. **a)** Intracellular flow cytometry using anti HA antibodies on total lung cell suspensions. Representative plots are gated on live $CD45^-$ cells. **b)** Immunohistochemistry using anti HA antibodies on section of early or late KP-OVA tumors. Scale bar: 200 µm.

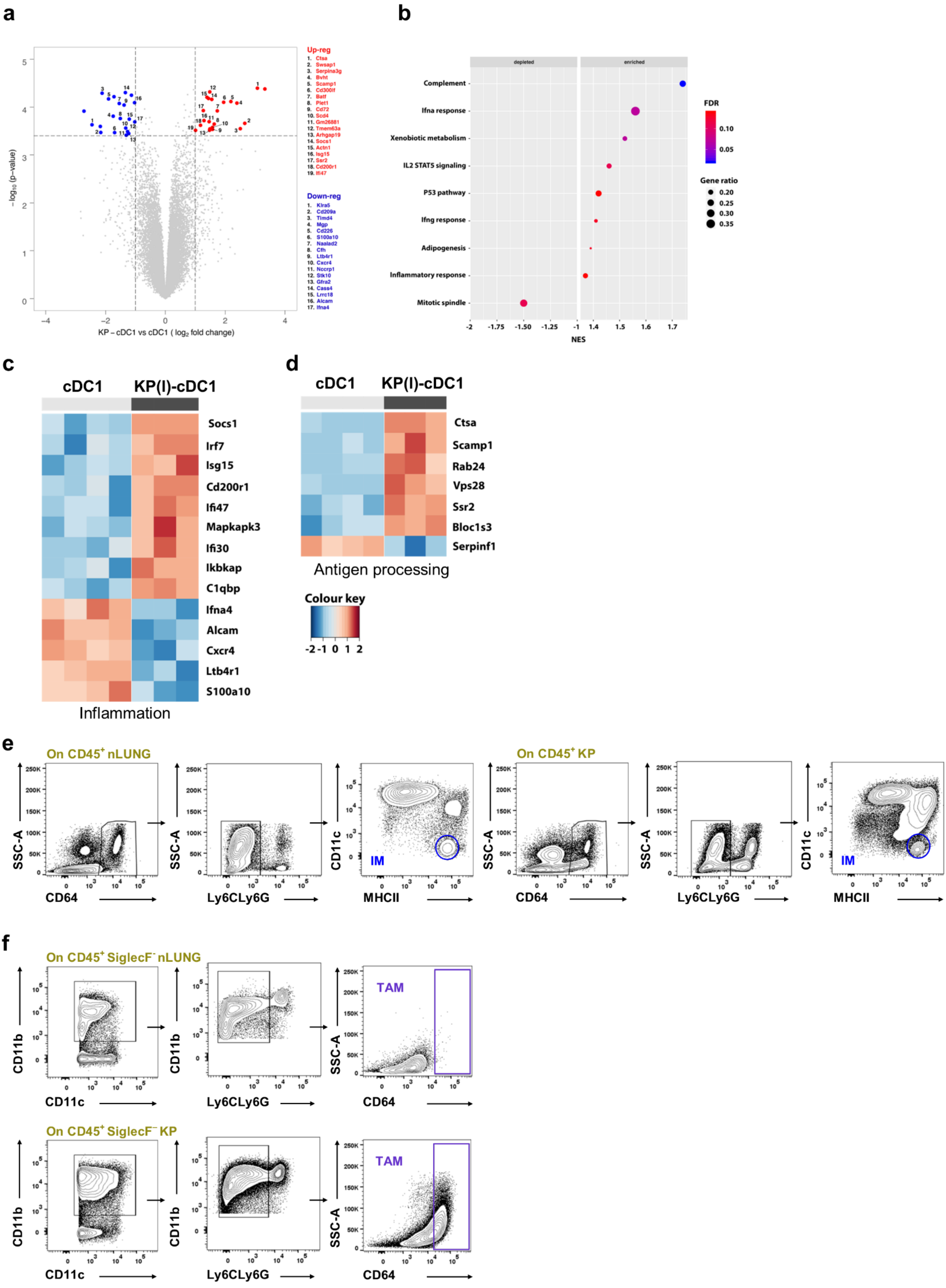
a
Up-reg
1. Ctsa
2. Swsap1
3. Serpina3g
4. Bvht
5. Scamp1
6. Cd300lf
7. Batf
8. Plet1
9. Cd72
10. Scd4
11. Gm26881
12. Tmem63a
13. Arhgap19
14. Socs1
15. Actn1
16. Isg15
17. Ssr2
18. Cd200r1
19. Ifi47
Down-reg
1. Klra5
2. Cd209a
3. Timd4
4. Mgp
5. Cd226
6. S100a10
7. Naalad2
8. Cfh
9. Ltb4r1
10. Cxcr4
11. Nccrp1
12. Stk10
13. Gfra2
14. Cass4
15. Lrrc18
16. Alcam
17. Ifna4
− log10 (p−value)
KP − cDC1 vs cDC1 (log2 fold change)
b
depleted
enriched
Complement
Ifna response
Xenobiotic metabolism
IL2 STAT5 signaling
P53 pathway
Ifng response
Adipogenesis
Inflammatory response
Mitotic spindle
FDR
0.10
0.05
Gene ratio
0.20
0.25
0.30
0.35
NES
c
cDC1
KP(I)-cDC1
Socs1
Irf7
Isg15
Cd200r1
Ifi47
Mapkapk3
Ifi30
Ikbkap
C1qbp
Ifna4
Alcam
Cxcr4
Ltb4r1
S100a10
Inflammation
d
cDC1
KP(I)-cDC1
Ctsa
Scamp1
Rab24
Vps28
Ssr2
Bloc1s3
Serpinf1
Antigen processing
Colour key
-2 -1 0 1 2
e
On CD45+ nLUNG
On CD45+ KP
SSC-A
CD64
Ly6CLy6G
CD11c
MHCII
IM
f
On CD45+ SiglecF- nLUNG
On CD45+ SiglecF- KP
CD11b
CD11c
Ly6CLy6G
SSC-A
CD64
TAM

Supplementary Figure 5. Modulation of genes involved in antigen processing and inflammation and downregulation in other tumor models.

a) Volcano plot showing differentially expressed genes in cDC1 from nLung or KP late tumor bearing lungs. Genes with a q-value<5% and a fold change ≥2 or ≤2 are identified as significantly modulated (red and blue, respectably), **b)** Dot plot of the significantly enriched and depleted gene sets from Gene Set Enrichment Analysis of cDC1 sorted from lungs with established KP tumors and residing in healthy lung tissues (normalized enrichment score NES>0). Dot color indicates statistical significance of the enrichment; dot size represents the fraction of genes annotated to each term. Gene sets are ranked in decreasing order based on the NES value. FDR: false discovery rate; NES: normalized enrichment score. **c, d)** Expression levels of genes differentially expressed (FDR<10%, absolute fold change >1.5) and annotated as interferon-alpha pathway (**c**) and antigen processing (**d**) from gene ontology in cDC1 from healthy and KP tumor bearing mice. **e, f)** Gating strategy used for the identification of Interstitial Macrophages (IM, **e**) and Tumor-associated macrophages (TAM, **f**) in nLung and KP-bearing lungs. In **a** to **d** differentially expressed genes were identified using the Significance Analysis of Microarray (SAM) algorithm, with a false discovery rate (FDR) ≤ 5% and absolute fold change larger than a selected threshold (e.g. ≥ 2).

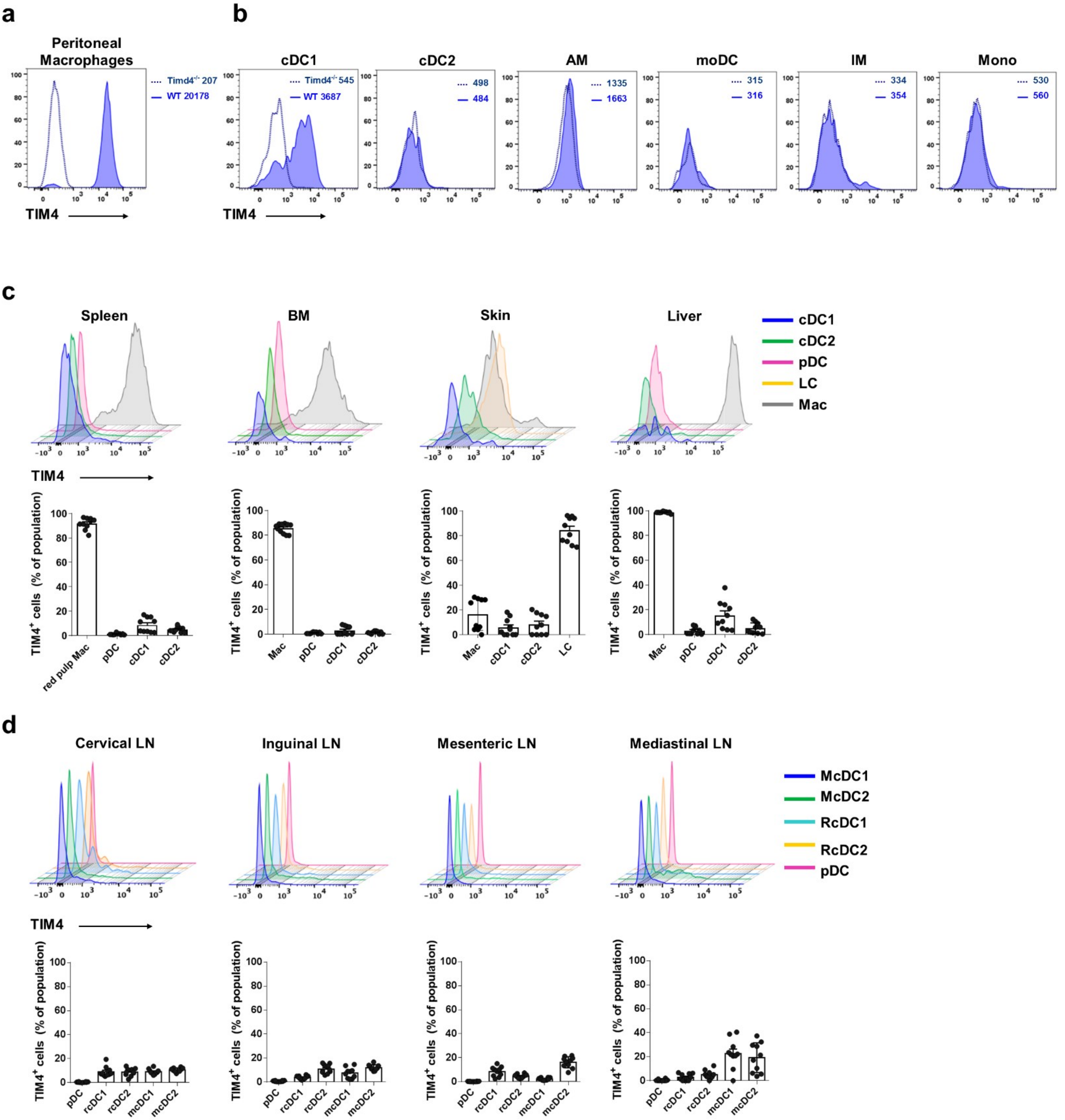
a
b
Peritoneal Macrophages
Timd4-/- 207
WT 20178
TIM4
cDC1
Timd4-/- 545
WT 3687
cDC2
498
484
AM
1335
1663
moDC
315
316
IM
334
354
Mono
530
560
c
Spleen
BM
Skin
Liver
cDC1
cDC2
pDC
LC
Mac
TIM4
TIM4+ cells (% of population)
red pulp Mac
pDC
cDC1
cDC2
Mac
LC
d
Cervical LN
Inguinal LN
Mesenteric LN
Mediastinal LN
McDC1
McDC2
RcDC1
RcDC2
pDC
TIM4
rcDC1
rcDC2
mcDC1
mcDC2

Supplementary Figure 6. Profile of TIM4 expression in DCs subsets and across tissues.
a) Specificity of TIM4 labeling evaluated on peritoneal macrophages from WT or Timd4$^{-/-}$ mice**. b)** Analysis of TIM4 expression by all phagocytes in nLung cDC1, cDC2, AM, moDC, IM and Mono. Cells from Timd4$^{-/-}$ lungs were used as control for specificity of labeling. **c)** Expression across the tissues and subsets, as indicated. Macrophages (Mac), conventional (cDCs) and plasmacytoid dendritic cells (pDC), Langerhans cells (LC) in spleen, BM, skin and liver. Representative histograms and quantification of the percentage of cells expressing TIM4 (on the FMO control) in organs of 10 individual mice. **d)** Flow cytometry analysis of TIM4 expression by subsets of dendritic cells in cervical, inguinal, mesenteric and mediastinal lymph nodes. Dendritic cell subsets analyzed: plasmacytoid DCs (pDC), resident conventional DC1 (rcDC1), resident conventional DC2 (rcDC2), migratory conventional DC1 (mcDC1) and migratory conventional DC2 (mcDC2). Representative histograms and quantification of the percentage of cells expressing TIM4 was calculated in organs of 10 individual mice. All data are expressed as mean ± SEM.

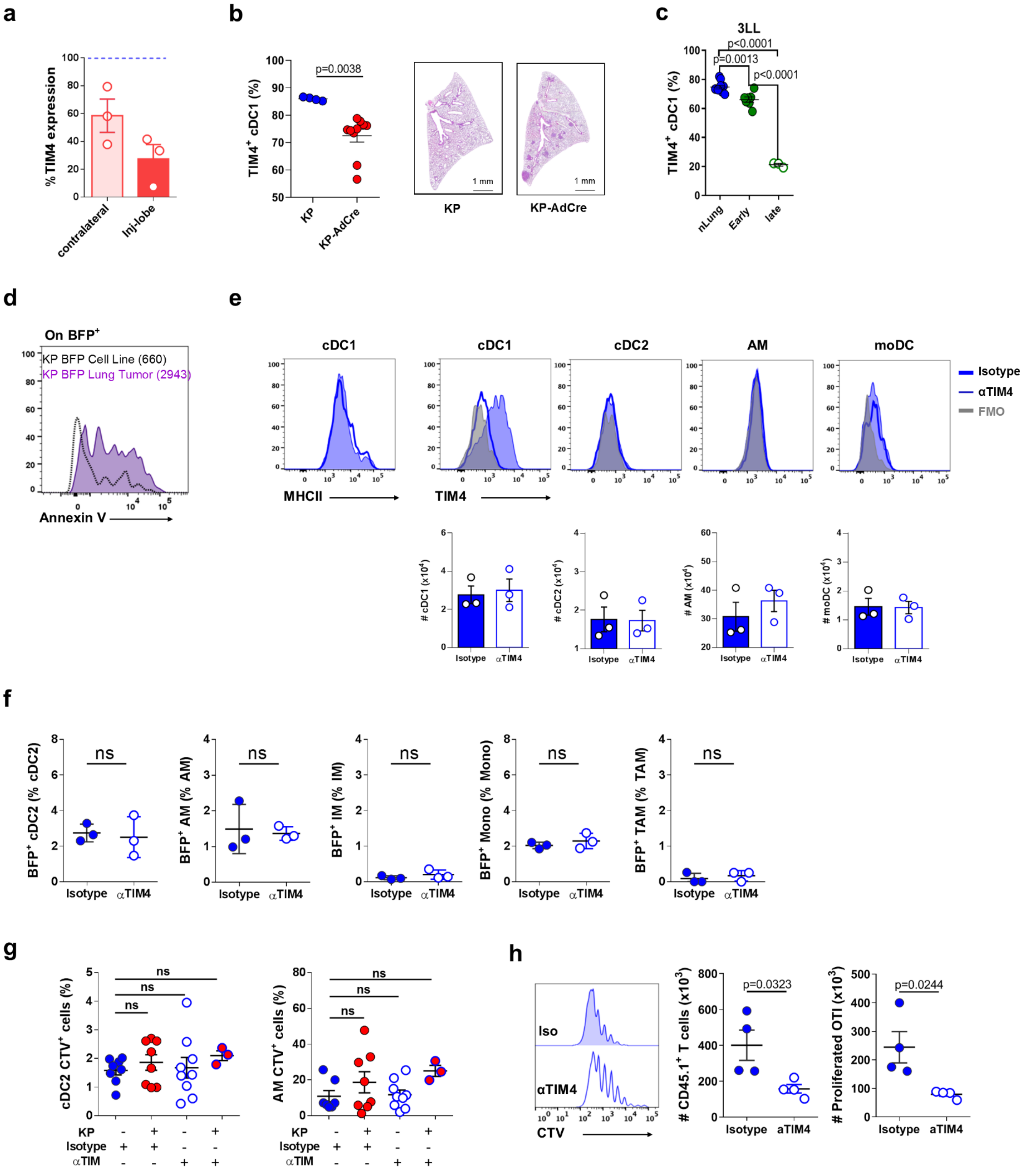

a
% TIM4 expression
contralateral
Inj-lobe
b
TIM4+ cDC1 (%)
p=0.0038
KP
KP-AdCre
1 mm
c
3LL
TIM4+ cDC1 (%)
p<0.0001
p=0.0013
p<0.0001
nLung
Early
late
d
On BFP+
KP BFP Cell Line (660)
KP BFP Lung Tumor (2943)
Annexin V
e
cDC1
cDC2
AM
moDC
Isotype
αTIM4
FMO
MHCII
TIM4
cDC1 (x10⁴)
cDC2 (x10⁴)
AM (x10⁴)
moDC (x10⁴)
f
ns
BFP+ cDC2 (% cDC2)
BFP+ AM (% AM)
BFP+ IM (% IM)
BFP+ Mono (% Mono)
BFP+ TAM (% TAM)
g
cDC2 CTV+ cells (%)
AM CTV+ cells (%)
KP
Isotype
αTIM
h
Iso
αTIM4
CTV
# CD45.1+ T cells (x10³)
p=0.0323
# Proliferated OTI (x10³)
p=0.0244
aTIM4

Supplementary Figure 7. TIM4 down-regulation in different tumor models, expression of PS by cancer cells, specificity of TIM4 blockade.

a) KP cells or PBS were injected orthotopically in the left lung lobe of healthy mice. After 28 days from tumor challenge lungs were harvested and TIM4 expression was analyzed in both injected or uninjected lobes, n=3 mice/group. **b)** TIM4 expression in cDC1 in KP autochthonous tumors. Tumors were induced by AdenoCre (AdCre) administration in KP mice, as described in M&M (KP-AdCre). Lungs from not treated (KP) and AdCre treated mice (KP-AdCre) were harvested 10 weeks after tumor induction for evaluation of TIM4 expression on whole lung tissues, KP n=4, KP-AdCre n=10. Graph represents 2 independent experiments. Statistical significance was assessed by two-tailed unpaired *t*-test. Representative hematoxylin/eosin sections of KP naïve and KP-AdCre are shown. **c)** TIM4 downregulation in lungs transplanted with 3LL. Mice were injected i.v. with Lewis Lung Carcinoma cells (3LL) and the lungs were harvested at day 14 (early) or 28 (late) after tumor implantation. Expression was compared to that found in control not challenged animals, nLung n= 7, Early n=7 and Late n=3. Statistical significance was assessed by one-way ANOVA followed by Tukey's post-test. **d)** Representative histograms showing Annexin V staining on KP-BFP cells in culture (Cell line) and in lung tissues 7 days after challenge (Lung Tumor). Numbers in parenthesis indicate MFI. **e)** Lungs were harvested from mice treated with anti-TIM4 antibodies or isotype control. Histograms show the indicated markers against FMO control in conventional dendritic cells (cDCs), AM and moDC. Graphs below indicate absolute numbers of each cell subset, one representative of two independent experiments with n=3 mice/group is shown **f)** Relative to Fig 4a. Uptake of KP-BFP cells by each phagocytic subset upon TIM4 blockade. The uptake was quantified 5 days after tumor challenge. Dot plot and quantification of one representative of two experiment with n=3 mice/group are shown. Significance was determined by two-tailed unpaired *t*-test, n=3 mice/group. **g)** Relative to Fig 4b. Percentage of cDC2 and AM associated to fluorescence 2hrs after challenge with apoptotic CTV-labelled thymocytes in nLung and KP bearing tumor lungs, treated or not with TIM4 blockade. Representative plots and quantification of two independent experiments are shown, Isotype n=7, Isotype KP(l)=9, αTIM4 n=8, αTIM4-KP(l) n=3. Statistical significance was assessed by one-way ANOVA followed by Tukey's post-test. **h)** Proliferation of transferred OTI in the lung of animals carrying early KP-OVA tumors and treated with α-TIM4 or isotype control antibodies was evaluated by CTV dilution. Representative histogram and quantification show the absolute numbers of adoptively transferred OTI and proliferated OTI (at least one cycle of proliferation) accumulated in the lungs. Significance was determined two-tailed unpaired *t*-test, n=4 mice/group. All data are expressed as mean±SEM. Raw data and statistical analysis are available in source data file.

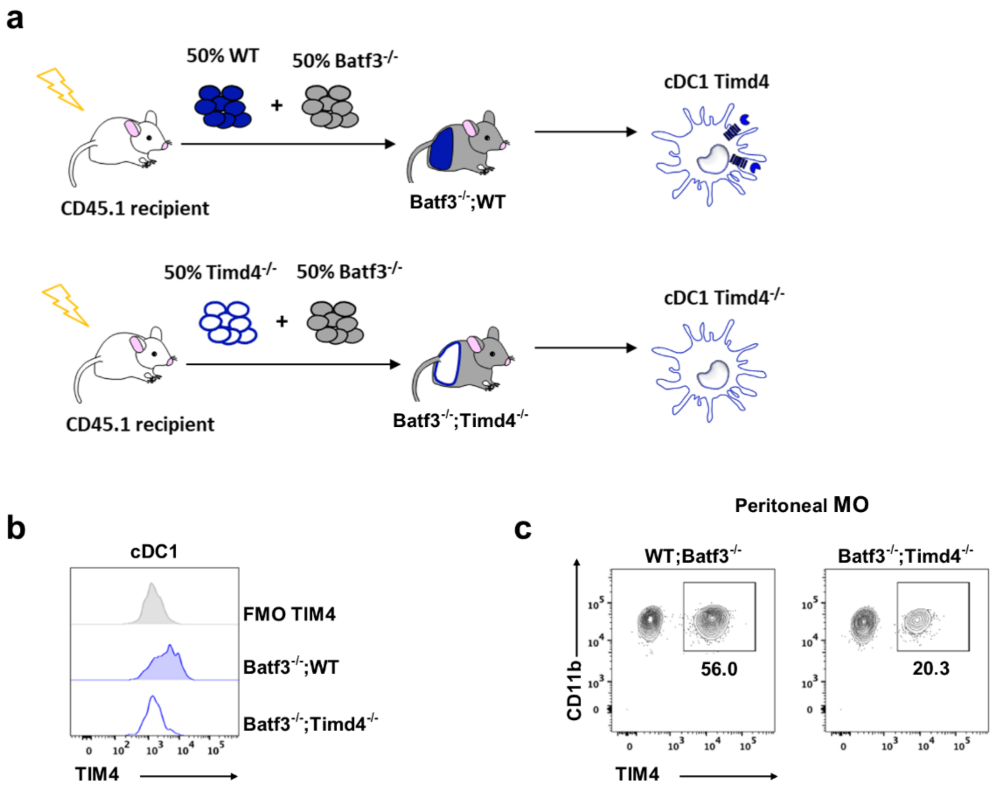

Supplementary Figure 8. Generation and validation of bone marrow chimeras.
a) Graphical representation of mixed bone marrow chimeras generation. Lethally irradiated CD45.1 recipient mice were transplanted with 1:1 ratio of WT;Batf3$^{-/-}$ or Timd4$^{-/-}$;Batf3$^{-/-}$ BM. **b, c)** Flow cytometry analysis of TIM4 expression by lung cDC1 (**b**) and peritoneal macrophages (**c**) of BM chimeric mice.

**Supplementary Table 1:** Differentailly expressed genes in cCD1 cells from tumor-bearing and healthy mice					
Gene symbol	*Description*	*EntrezID*	*Fold Change*	*p-value*	*q-value*
Isg15	ISG15 ubiquitin-like modifier	100038882	2,45	1,91E-04	0,0299
Gm26881	uncharacterized LOC105247101	105247120	2,80	2,85E-04	0,0299
Scamp1	3-hydroxyanthranilate 3,4-dioxygenase	107767	4,56	7,65E-05	0
3010003L21Rik	ubiquitin-conjugating enzyme E2Q family member 2	109163	2,76	2,01E-04	0,0299
Actn1	integrin alpha 1	109711	2,62	6,23E-05	0
Alcam	albumin	11658	-2,04	8,08E-05	0
Cd72	CD7 antigen	12517	2,98	2,91E-04	0,0299
Cfh	cripto, FRL-1, cryptic family 1	12628	-2,84	1,73E-04	0,0299
Socs1	suppressor of cytokine signaling 3	12703	2,71	6,58E-05	0
Cxcr4	chemokine (C-X-C motif) receptor 3	12767	-2,51	2,74E-04	0,0410
Gfra2	glial cell line derived neurotrophic factor family receptor alpha 1	14586	-2,34	3,56E-04	0,0467
Ifi47	interferon activated gene 204	15953	2,01	3,05E-04	0,0467
Ifna4	interferon alpha 11	15967	-2,03	2,04E-04	0,0410
Klra5	killer cell lectin-like receptor, subfamily A, member 4	16636	-5,48	2,34E-04	0,0410
Ltb4r1	lymphotoxin B	16995	-2,60	9,06E-05	0
Cd209a	CD209e antigen	170786	-4,47	3,39E-04	0,0467
Mgp	C-type lectin domain family 10, member A	17313	-3,35	1,55E-04	0,0299
Ctsa	PTPRF interacting protein, binding protein 2 (liprin beta 2)	19025	8,43	4,02E-05	0
S100a10	S100 calcium binding protein A1	20194	-3,24	3,40E-04	0,0467
Serpina3g	serine (or cysteine) peptidase inhibitor, clade A, member 3K	20715	5,67	2,82E-04	0,0299
Stk10	cAMP responsive element binding protein 3-like 3	20868	-2,40	3,24E-04	0,0410
Tmem63a	aurora kinase A	208795	2,80	4,81E-05	0
Cd226	zinc binding alcohol dehydrogenase, domain containing 2	225825	-3,29	6,07E-05	0
Nccrp1	sterile alpha motif domain containing 4B	233038	-2,48	3,86E-04	0,0467
Cd300lf	DnaJ heat shock protein family (Hsp40) member C28	246746	3,86	7,92E-05	0
Timd4	olfactory receptor 1371	276891	-4,34	5,12E-05	0
Cass4	cancer susceptibility candidate 1	320664	-2,31	1,78E-04	0,0299
A530064D06Rik	RIKEN cDNA 9830107B12 gene	328830	2,93	6,90E-05	0
Scd4	polycystic kidney disease 2-like 1	329065	2,97	2,66E-04	0,0299
2610524H06Rik	potassium channel tetramerisation domain containing 10	330173	10,03	4,18E-05	0
Batf	basic leucine zipper transcription factor, ATF-like	53314	3,33	1,20E-04	0,0251
Bvht	braveheart long non-coding RNA	545261	5,25	8,24E-05	0
Cd200r1	CD200 receptor 1	57781	2,25	2,40E-04	0,0299
Ssr2	signal sequence receptor, beta	66256	2,41	1,18E-04	0,0251
Swsap1	SWIM type zinc finger 7 associated protein 1	66962	6,29	2,18E-04	0,0299
2610301B20Rik	RIKEN cDNA 2610301B20 gene	67157	-6,60	1,21E-04	0,0299
Lrrc18	leucine rich repeat containing 18	67580	-2,20	5,64E-05	0
1600010M07Rik	RIKEN cDNA 1600010M07 gene	69781	-3,73	6,74E-05	0
Arhgap19	Rho GTPase activating protein 19	71085	2,78	3,04E-04	0,0467
9130008F23Rik	RIKEN cDNA 9130008F23 gene	71583	-2,53	4,97E-05	0
Naalad2	N-acetylated alpha-linked acidic dipeptidase 2	72560	-2,91	8,39E-05	0
Plet1	placenta expressed transcript 1	76509	3,10	2,27E-04	0,0299
2810429I04Rik	RIKEN cDNA 2810429I04 gene	76937	-4,52	2,53E-04	0,0410

Significance Analysis of Microarray (SAM) algorithm, with a q-value false discovery rate (FDR) < 0.05 and absolute fold change >2.

Supplementary Table 2: Gene signatures used to identify immune subsets and correlation p values (relative ton Fig 7)

Gene signature	Genes in signature	Ref paper	Pearson Corr coeff			p values		
			all stages	*stage I*	*stage II,III,IV*	*all stages*	*stage I*	*stage II,III,IV*
cDC1	CLEC9A, XCR1, CLNK, BATF3	1	0,416	0,506	0,336	9,84E-23	2,47E-19	1,53E-07
cDC2	CD1A, CD1C, CD1E, CD207 ,FCER1A, IRF4	2	0,394	0,443	0,372	2,92E-20	1,05E-14	4,88E-09
effector CD8	CD8A, CD8B,CD3E,PRF1,GZMB, GZMK	1	0,335	0,418	0,259	7,89E-15	4,44E-13	6,41E-05
NK	NCR1, NCR3, KLRB1, PRF1	1	0,357	0,46	0,263	1,06E-16	7,85E-16	4,93E-05
Mono-macro	LYZ, MAFB,CSF1R, CSF1R,CD300E, CD1A	3	0,404	0,407	0,408	2,41E-21	1,83E-12	1,06E-10
Neutro	CXCL8, CXCR2, S100A8, FCGR3B,CSFR3,FFAR2	2	0,237	0,196	0,277	6,31E-08	0,0011	1,81E-05
TAM	CD68,CSFR1, PPARy,CD64, TREM2, APOE, MARCO	3	0,336	0,345	0,33	7,69E-15	4,09E-09	2,59E-07

Pearson correlation coefficient in the *cor.test* function of R *stats* package with *two.sided* as alternative hypothesis

Supplementary Table 3. Primer sequences

Oligonucleotides	Source	Identifiers
Il12b reverse (ACAGGTGAGGTTCACTGTTTCT)	Integrated DNA technologies	N/A
Il 12a forward (GGCATCCAGCAGCTCCTCTC)	Integrated DNA technologies	N/A
Il 12a reverse (ACCCTGGCCAAACTGAGGTG)	Integrated DNA technologies	N/A
Ifna forward (GCA GAA GTC TGG AGA GCC CTC	Integrated DNA technologies	N/A
Ifna reverse (TGA GAT GCA GTG TTC TGG TCC)	Integrated DNA technologies	N/A
Ifnb forward (GCACTGGGTGGAATGAGTCT)	Integrated DNA technologies	N/A
Ifnb reverse (AGTGGAGAGCAGTTGAGGACA)	Integrated DNA technologies	N/A

Supplementary Table 4: List of antibodies

Flow Cytometry Antibodies	Dilution	Source	Identifiers
Purified anti-mouse CD16/32 Antibody (93)	1:100	BioLegend	Cat# 101302, RRID:AB_312801
7AAD	5 µl	BioLegend	Cat# 420404
Annexin-V BV421	5 µl	BioLegend	Cat# 640923, RRID:AB_2562965
Anti-mouse CD3e (145-2C11) A488	1:200	BioLegend	Cat# 100321, RRID:AB_389300
Anti-mouse CD3e (145-2C11) Biotin	1:100	BD Biosciences	Cat# 553060, RRID:AB_394593
Anti-mouse CD4 (GK1.5) BV785	1:200	BioLegend	Cat# 100453, RRID:AB_2565843
Anti-mouse CD8a (53-6.7) APC	1:200	BioLegend	Cat# 100711, RRID:AB_312750
Anti-mouse CD11b (M1/70) BV421	1:500	BD Biosciences	Cat# 562605, RRID:AB_11152949
Anti-mouse CD11c (N418) A647	1:200	BioLegend	Cat# 117314, RRID:AB_492850
Anti-mouse CD19 (1D3) Biotin	1:100	BD Biosciences	Cat# 553784, RRID:AB_395048
Anti-mouse CD24 (M1/69) PE/Dazzle™594	1:800	BioLegend	Cat# 101837, RRID:AB_2566731
Anti-mouse CD31 (390) Biotin	1:100	BioLegend	Cat# 102404, RRID:AB_312899
Anti-mouse CD45 (30-F11) APC/Cy7	1:200	BioLegend	Cat# 103115, RRID:AB_312980
Anti-mouse CD45.1 (A20) PE	1:200	Thermo Fisher Scientific	Cat# 12-0453-81, RRID:AB_465674
Anti-mouse CD45.1 (A20) APC	1:300	BioLegend	Cat# 110714, RRID:AB_313503
Anti-mouse CD45.2 (104) FITC	1:100	Thermo Fisher Scientific	Cat# 11-0454-82, RRID:AB_465061
Anti-mouse CD45R/B220 (RA3-6B2) APC-eFluor780	1:200	Thermo Fisher Scientific	Cat# 47-0452-82, RRID:AB_1518810
Anti-mouse CD49b (DX5) APC	1:200	BioLegend	Cat# 108909, RRID:AB_313416
Anti-mouse CD64 (X54-5/7.1) BV711	1:200	BioLegend	Cat# 139311, RRID:AB_2563846
Anti-mouse CD103 (2E7) PE	1:200	BioLegend	Cat# 121405, RRID:AB_535948
Anti-mouse CD326/EpCAM (G8.8) BV605	1:200	BioLegend	Cat# 118227, RRID:AB_2563984
Anti-mouse F4/80 (T45-2342) PE-CF594	1:100	BD Biosciences	Cat# 565613, RRID:AB_2734770
Anti-mouse H-2Kb bound to SIINFEKL (25-D1.16) PE	1:200	BioLegend	Cat#141603, RRID:AB_10897938
Anti-mouse IFN-γ (XMG1.2) PE	1:100	BioLegend	Cat# 505808, RRID:AB_315402
Anti-mouse IL-12p40 (C15.6)	1:100	BD Biosciences	Cat# 554479, RRID:AB_395420

Anti-mouse Ly-6G/Ly-6C (RB6-8C5) Biotin	1:200	BD Biosciences	Cat# 553124, RRID:AB_394640
Anti-mouse Ly6C (HK1.4) A488	1:100	BioLegend	Cat# 128022, RRID:AB_10639728
Anti-mouse Ly6G (1A8) PE	1:50	BioLegend	Cat# 127608, RRID:AB_1186099
Anti-mouse MerTK (DS5MMER) PE-Cy7	1:200	Thermo Fisher Scientific	Cat# 25-5751-82, RRID:AB_2573466
Anti-mouse MHCII (I-A/I-E) (M5/114.15.2) BV711	1:200	BD Biosciences	Cat# 563414, RRID:AB_2738191
Anti-mouse MHCII (I-Ab) (AF6-120.1) A488	1:200	BioLegend	Cat# 116410, RRID:AB_493140
Anti-mouse NK1.1 (PK136) FITC	1:200	Thermo Fisher Scientific	Cat# 11-5941-82, RRID:AB_465318
Anti-mouse NKp46 (29A1.4) eFluor450	1:100	Thermo Fisher Scientific	Cat# 48-3351-82, RRID:AB_10557245
Anti-mouse SiglecF (E50-2440) BB515	1:200	BD Biosciences	Cat# 564514, RRID:AB_2738833
Anti-mouse SiglecF (IRNM44N) PerCP-eFluor 710, eBioscience™	1:200	Thermo Fisher Scientific	Cat# 46-1702-82, RRID:AB_2573724
Anti-mouse SiglecH (551) PercP-Cy5.5	1:200	BioLegend	Cat# 129614, RRID:AB_10643995
Anti-mouse TER-119 (TER-119) Biotin	1:200	BioLegend	Cat# 116203, RRID:AB_313704
Anti-mouse TIM4 (F31-5G3) PE	1:400	BioLegend	Cat# 129905, RRID:AB_1227799
Anti-mouse TCR $V\alpha2$ (B20.1) PerCP/Cyanine5.5	1:200	BioLegend	Cat# 127814, RRID:AB_1186116
Anti-mouse TCR V beta 5.1/5.2 (MR9-4) eFluor 450, eBioscience™	1:200	Thermo Fisher Scientific	Cat# 48-5796-82, RRID:AB_2574075
Anti-mouse XCR1 (ZET) BV650	1:200	BioLegend	Cat# 148220, RRID:AB_2566410
Pro5® MHC H- 2KbPentamers PE	5 µl	Proimmune	Cat# F093-2A-E
Streptavidin APC-Cy7	1:200	BioLegend	Cat#405208
Anti-HA High Affinity (3F10)	1:50	Roche	Cat# 11867423001, RRID:AB_10094468
Goat anti-Rat IgG (H+L) Cross-Adsorbed Secondary Antibody, Alexa Fluor 647	1:200	Thermo Fisher Scientific	Cat# A-21247, RRID:AB_141778
Depleting/blocking antibodies			
Anti-mouse TIM4 (RMT4-53)		BioXCell	Cat# BE0171, RRID:AB_2687695
Anti-mouse CD8 (YTS 169.4)		BioXCell	Cat# BE0117, RRID:AB_10950145
Rat IgG2a Isotype control (2A3)		BioXCell	Cat# BE0089, RRID:AB_1107769
Antibodies for immunohistochemistry			
Anti-mouse CD8 (4SM15)	1:100	Thermo Fisher Scientific	Cat# 14-0808-82, RRID:AB_2572861
Anti-HA High Affinity (3F10)	1:50	Roche	Cat# 11867423001, RRID:AB_10094468

Supplementary Table 5: Flow cytometry panels identification

Tim4 expression in myeloid cells	
CD45	APCCy7
CD11c	APC
MHCII	AF700
SiglecF	PerCPCy5.5
CD64	BV605
CD11b	BV421
CD24	PEDazzle
XCR1	BV650
F4/80	AF488
TIM4	PE
Live/Dead fixable	Aqua
TIM4 expression in lung (Neu/Mono/Eo)	
CD45	APCCy7
CD11b	BV421
SiglecF	PerCPCy5.5
Ly6G	FITC
Ly6C	BV570
CD11c	APC
TIM4	PE
Live/Dead fixable	Aqua
TIM4 expression in lung (T cell)	
CD45	APCCy7
CD3	FITC
CD4	BV785
CD8	APC
TIM4	PE
Live/Dead fixable	Aqua
TIM4 expression in peritoneum	
F4/80	AF488
MHCII	AF700
CD11b	PerCPCy5.5
CD64	BV605
TIM4	PE
Live/Dead fixable	Aqua
Isolation of myeloid cells	
MHCII	BV711
CD11c	APC
SiglecF	PerCPCy5.5
CD64	BV605
CD11b	BV421
CD24	PEDazzle
XCR1	BV650
F4/80	AF488
Uptake of cell associated tumor antigens	
CD45	APCCy7
CD11c	APC

MHCII	AF488
SiglecF	PerCPCy5.5
XCR1	BV650
	CTV/BFP
Live/Dead fixable	Aqua
In-vivo cross-presentation	
CD11c	APC
MHCII	AF488
SiglecF	PerCPCy5.5
CD103	BV421
XCR1	BV650
25D.1	PE
Live/Dead fixable	Aqua
In-vivo T cell activation	
CD45	APCCy7
CD3	PerCPCy5.5
CD8	APC
Pentamers	PE
Live/Dead fixable	Aqua
OTI T cell proliferation assay	
CD8	APC
	CFSE
Live/Dead fixable	Aqua
Proliferation of transferred CD45.1 OT-I T cell	
CD3	AF488
CD8	APC
CD45.1	PE
	CTV
Live/Dead fixable	Aqua
IFN-γ production by endogenous CD8 T cell	
CD45	APCCy7
CD3	AF488
CD8	APC
Vβ5	BV421
Vα2	PerCPCy5.5
IFN-γ	PE
Live/Dead fixable	Aqua
IFN-γ production by transferred OTI T cell	
CD8	AF488
CD45.1	APC
IFN-γ	PE
Live/Dead fixable	Aqua

Supplementary References (relative to Supplementary Table 2)

1 Bottcher, J. P. *et al.* NK Cells Stimulate Recruitment of cDC1 into the Tumor Microenvironment Promoting Cancer Immune Control. *Cell* **172**, 1022-1037 e1014, doi:10.1016/j.cell.2018.01.004 (2018).

2 Zilionis, R. *et al.* Single-Cell Transcriptomics of Human and Mouse Lung Cancers Reveals Conserved Myeloid Populations across Individuals and Species. *Immunity* **50**, 1317-1334 e1310, doi:10.1016/j.immuni.2019.03.009 (2019).

3 Lavin, Y. *et al.* Innate Immune Landscape in Early Lung Adenocarcinoma by Paired Single-Cell Analyses. *Cell* **169**, 750-765 e717, doi:10.1016/j.cell.2017.04.014 (2017).
